# Rapid high-throughput analysis of DNaseI hypersensitive sites using a modified Multiplex Ligation-dependent Probe Amplification approach

**DOI:** 10.1186/1471-2164-10-412

**Published:** 2009-09-04

**Authors:** Thomas Ohnesorg, Stefanie Eggers, Wouter N Leonhard, Andrew H Sinclair, Stefan J White

**Affiliations:** 1 Murdoch Children's Research Institute and Department of Paediatrics, University of Melbourne, Royal Children's Hospital, Parkville, VIC, Australia; 2 Center for Human and Clinical Genetics, Leiden University Medical Center, Leiden, The Netherlands

## Abstract

**Background:**

Mapping DNaseI hypersensitive sites is commonly used to identify regulatory regions in the genome. However, currently available methods are either time consuming and laborious, expensive or require large numbers of cells. We aimed to develop a quick and straightforward method for the analysis of DNaseI hypersensitive sites that overcomes these problems.

**Results:**

We have developed a modified Multiplex Ligation-dependent Probe Amplification (MLPA) approach for the identification and analysis of genomic regulatory regions. The utility of this approach was demonstrated by simultaneously analysing 20 loci from the ENCODE project for DNaseI hypersensitivity in a range of different cell lines. We were able to obtain reproducible results with as little as 5 × 10^4 ^cells per DNaseI treatment. Our results broadly matched those previously reported by the ENCODE project, and both technical and biological replicates showed high correlations, indicating the sensitivity and reproducibility of this method.

**Conclusion:**

This new method will considerably facilitate the identification and analysis of DNaseI hypersensitive sites. Due to the multiplexing potential of MLPA (up to 50 loci can be examined) it is possible to analyse dozens of DNaseI hypersensitive sites in a single reaction. Furthermore, the high sensitivity of MLPA means that fewer than 10^5 ^cells per DNaseI treatment can be used, allowing the discovery and analysis of tissue specific regulatory regions without the need for pooling. This method is quick and easy and results can be obtained within 48 hours after harvesting of cells or tissues. As no special equipment is required, this method can be applied by any laboratory interested in the analysis of DNaseI hypersensitive regions.

## Background

Open chromatin is a characteristic of genomic loci with regulatory functions. These regions are preferentially digested by DNaseI [1], and the identification of DNaseI hypersensitive sites is frequently used to identify and analyse regulatory regions such as promoters, enhancers and silencers [2,3]. However, currently available methods have significant limitations. A commonly used approach involves Southern blotting, but this is time consuming, usually requires radioactivity and is limited to short stretches of DNA. Several PCR-based methods have been described [4,5], but these do not readily allow multiplexing. Recent reports of large scale analysis of DNaseI hypersensitive sites have used either microarrays [6-9] or deep sequencing [9,10]. Whilst valuable for genome wide analysis, the costs involved are a limiting factor for many applications (such as comparing different developmental stages or tissues). Another disadvantage of those methods is that they usually require many millions of cells. For *ex vivo *studies, this might require extensive pooling of tissues, meaning that these methods are not suitable for all applications.

Multiplex Ligation-dependent Probe Amplification (MLPA) was originally developed to detect deletions and duplications in genomic DNA [11], and has become popular in diagnostic settings for a range of disorders [12,13]. It has since been modified for several other applications as well, including methylation analysis [14], mRNA expression analysis [15], identifying copy number variation in normal populations [16,17], genotyping of mouse models [18,19] and measuring the efficiency of Cre-mediated recombination in mouse models [20]. The principle advantages of this method are the sensitivity and multiplexing potential. It can be used to analyse up to 50 genomic loci with as little as 20 ng genomic DNA in a single reaction. Furthermore, the only equipment that is required is a thermocycler and DNA sequencer, readily available to most researchers.

We describe here a quick and straightforward protocol for analysing DNaseI hypersensitive sites. This is demonstrated by the analysis of 20 different loci in a single reaction, based on data published by the ENCODE consortium [21].

## Results

### Probe design

**Figure 1:**
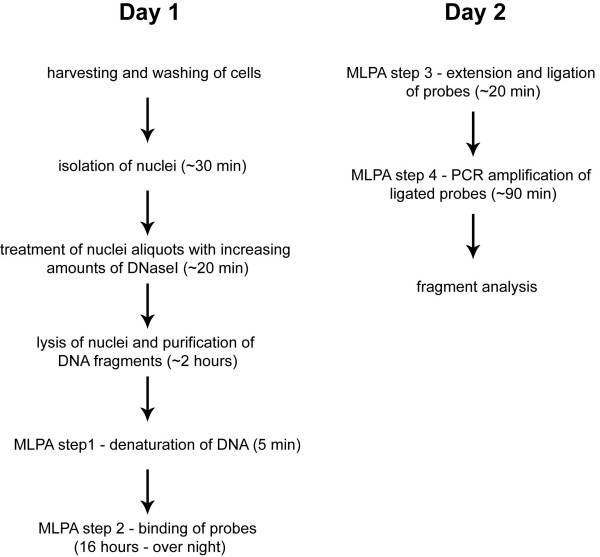
**Method overview**. Schematic overview of the steps involved in DNaseI-MLPA, including approximate times required for each step when using four different cell preparations with three different DNaseI concentrations. Results can be obtained in less than 48 hours.

Figure 1 outlines the protocol used in this study. To examine the general feasibility of our approach, we designed 11 probes to cover randomly chosen DNaseI hypersensitive sites in HeLa cells as published by the ENCODE consortium [21]. In addition, nine probes were designed in regions that showed no evidence of DNaseI hypersensitivity. To be able to cover larger genomic regions and to give greater flexibility in probe design we employed the recently developed extension MLPA [20]. All 20 probes were combined in a single mix, and could be differentiated from each other on the basis of length (the final product length range was 94-207 bp).

### Nuclei isolation and DNaseI treatment

**Figure 2:**
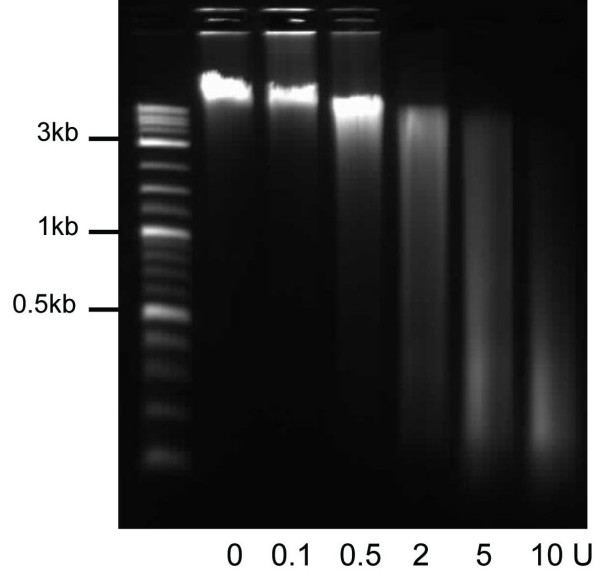
**Example of DNaseI digest**. Typical result of the analysis of DNaseI treated samples by agarose gel electrophoresis. The untreated sample and those digested with 0.5 and 2 units of DNaseI were used in subsequent experiments. L = DNA ladder.

We tested different methods for the isolation of nuclei, with the Ne-Per Kit (Thermo Scientific) found to be most suitable. Although intended for the isolation and separation of nuclear and cytoplasmic proteins, it proved to be useful for the isolation of nuclei. Another obstacle to overcome was the isolation of the genomic DNA. This approach requires efficient recovery of very long as well as very short fragments of genomic DNA. Unfortunately, DNA obtained using the standard method for this task, phenol-chloroform purification, is not suitable for use with MLPA, as traces of phenol are known to interfere with the enzymatic reactions. Instead we used the HighPure PCR Purification Kit (Roche), which, according to the manufacturer, is suitable for the isolation of fragments of up to 50 kb. As expected, in our experiments the recovery of DNA from the untreated and therefore most intact sample, was often least efficient, however, the quality and quantity of the DNA was still sufficient for MLPA analysis. Figure 2 shows a typical example of digested DNA obtained after treating isolated nuclei with increasing amounts of DNaseI and purification.

### Analysis of DNaseI hypersensitive sites in HeLa cells

**Figure 3:**
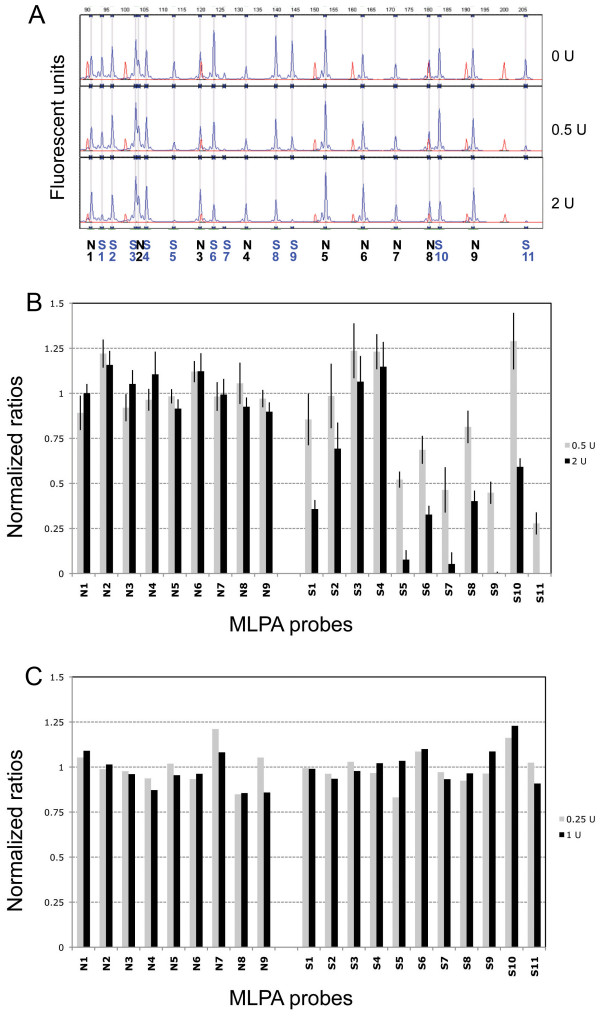
**MLPA on DNA from HeLa cells**. A) Representative MLPA peak patterns obtained from nuclei isolated from HeLa cells and subsequent treatment with 0, 0.5 and 2 units of DNaseI. Red peaks represent size standards, blue peaks the signals from the probes after PCR amplification. Black numbers show probes designed to bind to non-sensitive regions, blue numbers to sensitive regions for HeLa cells as published by the ENCODE consortium. B) Analysis of experiment shown in 3A. Data were obtained from two independent experiments and two technical replicates (n = 4). Results shown as mean ± SD. C) Analysis of data derived from DNaseI treatment of naked DNA isolated from HeLa cells. In this case DNaseI digestion was with either 0.25 units or 1 unit DNaseI for 1 minute on ice, as this DNA was far more susceptible to degradation than DNA in intact nuclei. Probes are grouped into sensitive and non-sensitive, and then ordered according to their length. N: non-sensitive, S: sensitive for HeLa cells as published by the ENCODE consortium.

We next used the same probe mix to analyse the DNaseI hypersensitivity of DNA within intact nuclei of HeLa cells. Figure 3A shows typical peak patterns obtained using nuclei aliquots digested with increasing amounts of DNaseI. Several peaks show significantly decreased peak height with increased DNaseI concentration, while others remain virtually unchanged. Nine of 11 probes targeting previously described DNaseI hypersensitive sites in HeLa cells show a clear decrease in normalized peak heights (defined as < 75% of the equivalent peak in undigested DNA), whereas all nine probes targeting non-sensitive loci show no significant decrease (figure 3B). These results were highly reproducible, with the technical and biological replicates giving an r^2 ^> 0.9.

We also tested the same probe mix on DNA directly isolated from HeLa cells. As can be seen in Figure 3C, there are no significant changes in normalized peak heights with increasing DNaseI digestion. This was expected as these DNA samples should have no chromatin structure, and all cuts by DNaseI should therefore occur in a random fashion.

### Comparison of results using other cell lines

**Figure 4:**
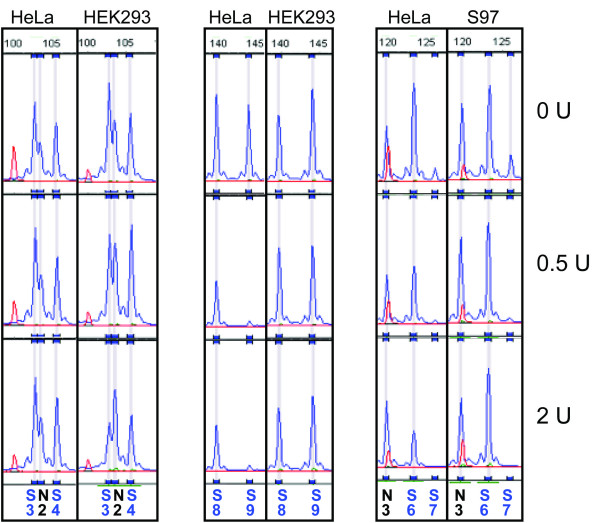
**Differences in DNaseI sensitivity between the tested cell lines**. The majority of probes gave similar peak patterns in all tested cell lines, with the exceptions shown here. As can be seen on the left, the two regions not showing sensitivity in HeLa cells are sensitive in HEK293 cells. On the other hand, region S9 in HEK293 and S6 in S97 cells show no sign of sensitivity in contrast to HeLa cells.

We then analysed the same 20 loci for DNaseI hypersensitivity in other cell lines. For this comparison we used HEK293, C28 and S97 cells. While the results for C28 cells were very similar to those of HeLa cells (data not shown), we could identify differences in sensitivity for several probes in HEK293 and S97 cells. As shown in Figure 4, some probes identifying hypersensitive sites in HeLa cells stay unchanged in the other cell lines (probes S9 in HEK293 cells, S6 in S97 cells) and two of the probes not showing the expected sensitivity in HeLa cells (S3 and S4) do show sensitivity in HEK293 cells.

### Determining the minimum cell number

**Figure 5:**
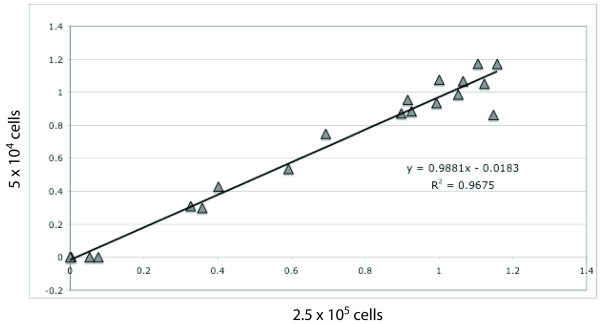
**Comparison of results obtained using different cell numbers**. Correlation of results obtained from experiments with 5 × 10^4 ^(n = 1) and 2.5 × 10^5 ^(n = 4) HeLa cells per DNaseI treatment aliquot.

We also tried to determine the minimum cell number that could be used with this approach. As we were able to obtain robust results and sufficient DNA for several replicates from aliquots containing nuclei from about 2.5 × 10^5 ^cells, we estimated the lower limit of required cells to be around 5 × 10^4 ^(theoretically ~300 ng DNA). To confirm this number, we used aliquots of 5 × 10^4 ^HeLa cells and treated them as described above and compared the results with those obtained from experiments using 2.5 × 10^5 ^cells per aliquot. Figure 5 shows the comparison of results from 5 × 10^4 ^and 2.5 × 10^5 ^cells (r^2 ^= 0.97).

## Discussion

We describe here a simple technique that allows the rapid analysis of many DNaseI hypersensitive sites using little starting material. For a proof of principle we chose loci that had been analysed by both array analysis and deep sequencing as part of the ENCODE project [21].

We used 20 probes in one reaction (11 sensitive, 9 non-sensitive in HeLa cells according to ENCODE data). Nine of 11 of the probes targeted to hypersensitive sites showed the expected decrease in peak height for HeLa cells. The fact that two sensitive sites did not show the expected drop in peak heights is probably due to different growth conditions, differences in passage numbers or to our batch of HeLa cells being different from the one used in the ENCODE study rather than to limitations of our method. This is supported by the high correlation observed for both technical and biological replicates (r^2 ^> 0.9) and the fact that those two probes showed sensitivity in HEK293 cells.

We also examined three other cell lines, in order to examine the cell line specificity of our experimental approach. We could show clear differences in hypersensitivity for some loci compared to HeLa cells. Again, the conclusion that these are cell type-specific differences is supported by the concordance of replicate experiments (r^2 ^> 0.9).

To determine suitable DNaseI digestion conditions for our method, a range of different DNaseI concentrations and incubation conditions were tested. Incubating the digestion reactions with the indicated DNaseI amounts at room temperature for 20 minutes gave more reproducible results than, for example, using less DNaseI and incubating at 37°C or incubations on ice (data not shown). By analysing the digested DNA on an agarose gel it was possible to identify the most appropriate conditions. Most of our experiments were carried out using nuclei from 2.5 × 10^5 ^cells per DNaseI digestion aliquot, providing sufficient DNA for several technical replicates. However, in the case of HeLa cells this number could be reduced to only 5 × 10^4 ^cells, still providing highly reproducible results as shown by an r^2 ^of 0.97 when comparing the results of the experiments with both cell numbers. Furthermore, we found that two different DNaseI concentrations and an undigested control are sufficient to analyse hypersensitive sites, which reduces the amount of total starting material required to about 1.5 × 10^5 ^cells.

Although we have used 20 probes in this study, there is the potential to significantly increase the degree of multiplexing. Reducing the interprobe spacing to 3 bp would allow up to 40 loci to be examined. Indeed, by designing probes that can be labelled with different fluorophores [22,23] it would be possible to analyse > 100 loci in a single reaction.

## Conclusion

We describe here a rapid and accurate method for assaying DNaseI hypersensitive sites. In contrast to genome-wide approaches such as deep sequencing or microarray analysis, we consider the primary strength of this approach to be when < 100 genomic loci are being analysed. As all loci are analysed in a single reaction, relatively little starting material is required. We have shown that < 10^5 ^cells per DNaseI treatment can be used, which will allow the study of e.g. embryonic organ development without extensive pooling. The protocol is straightforward and results can be easily obtained from many samples within 48 hours. Finally, using this method makes it possible to also detect heterozygous and homozygous deletions and duplications of the examined regions, which is an artifact that is known to occur in cultured cells.

## Methods

### Cell culture

HEK293 (human embryonic kidney cells), C28 (chondrogenic cells), HeLa (cervical cancer cells) and S97 (dermal fibroblasts) were grown in DMEM containing 10% FBS and supplemented with L-glutamine in T-25 flasks and incubated at 37°C containing 5% CO_2 _in a humidified atmosphere.

### Cell harvest and isolation of nuclei

After reaching 95-100% of confluence, cells were washed with PBS and harvested using 1 ml of 0.025% Trypsin-EDTA. After 5-10 min incubation at 37°C, cells were washed with 2 ml of 10% FBS containing DMEM and carefully resuspended in a 1.5 ml Eppendorf tube in cold PBS and placed on ice. To limit changes in chromatin structure during treatment, nuclei isolation was performed using 2.5 × 10^5 ^to 10^6 ^cells as soon as possible.

For isolation of nuclei, the NE-PER Kit (Thermo Scientific) was used with the following modifications. To prevent nuclei from excessive clumping and releasing DNA, twice the recommended volumes of the solutions were used and resuspension of cells/nuclei was carried out by carefully pipetting up and down rather than vortexing. Centrifugation steps were carried out at 250 × g at 4°C.

### DNaseI treatment of nuclei

Isolated nuclei were washed in 500 μl cold DNaseI buffer containing 2% glycerol and carefully resuspended in 75 μl DNaseI buffer containing 2% glycerol. 25 μl aliquots of the nuclei suspension were added to 2 ml Eppendorf tubes containing one control without DNaseI and increasing amounts (0.5 - 2 units) of DNaseI (Promega) in 50 μl DNaseI buffer containing 2% glycerol. The solutions were mixed by carefully flicking the tubes and incubated for 20 min at room temperature (23°C).

### Isolation and purification of DNaseI treated genomic DNA

DNaseI treated nuclei were lysed by adding 250 μl of nuclei lysis buffer (200 mM NaCl, 150 mM Tris HCl pH 8, 10 mM EDTA pH 8, 0.2% SDS) containing 50 μg of Proteinase K and incubated for 45 min at 55°C. 20 μg of RNaseA (Sigma) was added and incubated for 30 min at 37°C.

The DNaseI treated DNA was isolated and purified using the HighPure PCR Purification Kit (Roche) according to the manufacturer's instructions. DNA was eluted with 50 μl elution buffer provided with the kit. Purified DNA was checked for quality and degree of DNaseI digestion by agarose gel electrophoresis. DNA concentrations were measured using the NanoDrop.

### MLPA and fragment analysis

The MLPA probe mix was created by combining each MLPA oligonucleotide in table 1 to a final concentration of 4 nM. The extension MLPA was performed essentially as described [20]. 100-200 ng of DNaseI-treated DNA of each sample was denatured at 98°C for 5 min, and allowed to cool to room temperature. 1.5 μl MLPA buffer (MRC-Holland, the Netherlands) and 1.5 μl MLPA probe mix were added to each sample, denatured at 95°C for 1 min then hybridized overnight at 60°C. The following morning a ligation and elongation step was carried out at 54°C by adding 3 μl ligase buffer A, 3 μl ligase buffer B, 1 μl Ligase-65 (all MRC Holland, the Netherlands), 2 mM dNTPs, 1 U Stoffel Taq polymerase (Applied Biosystems) and H_2_O to a final volume of 40 μl. After 15-20 min the ligase was inactivated by heating to 95°C for 5 min. The PCR reaction consisted of 5 μl of ligated mix added to 2 μl SALSA PCR buffer, 1 μl SALSA enzyme buffer, 1 μl SALSA PCR primer, 0.25 μl SALSA polymerase (all MRC-Holland, the Netherlands) and 15.75 μl H_2_O. The PCR reaction started with a 1 min denaturation step at 95°C, followed by 35-38 cycles of amplification (each cycle consisting of 95°C for 30 sec, 58°C for 30 sec, 72°C for 30 sec) and concluded with 20 min at 72°C.

**Table 1:** Probe sets used in this study.

**Name**	**Probe1**	**Probe2**	**Genomic location**	**Product size**	**Gap size**
					

N1	1AAGACAGAGTCAGCACCAAGCAACCTG	GAGCGGCTGCTTCTTTCTCTCTTGC2	chr2:234,440,746-234,440,797	94	-

N2	1GAGCAGCACTTAGTACACAGAGGCCTCTG	CCAGGATTGCAGAAGGCTTGCAGAGG2	chrX:153,258,524-153,258,587	106	9

N3	1GACCTTACTTTGATGAAGGCAGTTCTGC	CATGGGTGCCACGGTTTGAATGTATC2	chr21:33,073,083-33,073,161	121	25

N4	1CCCAGTGACCTACAGTAGAACTTTTCTGTGTCC	GCTGTTCTCCGTGCCTATCACCTGTTAAAGG2	chr2:234,444,778-234,444,867	132	26

N5	1CAAATAGTCGAGTGGTACCTGTTCAGCC	GACAGAACTAGGAAACAAATACCTCCTCATTCTATATGGC2	chr5:131,655,909-131,656,020	154	44

N6	1GACAGAAAATGCAGTCCAGTTGGTACAAGC	CTCTCTCAGGGCTGCTTCATGAACTTAC2	chr21:33,778,023-33,778,144	164	64

N7	1GCTGTTCAGCATTGGTGTAAGTTCTGATTCC	CTCCAGACACCTGAGCCAAGAGAAAGATT2	chr21:33,068,576-33,068,705	172	70

N8	1CTAAGGTGGCCATGCTTCTCTGGATTTGC	CAGCTCATCCCGCGTCGATTCCTGGAAGTGTTATC2	chr5:131,660,201-131,660,338	180	74

N9	1GGAAAGAGCAGGAGAAAGGGAATCTTGG	GTCTCTTCAGCTTGTGGGAACAAACGAG2	chr7:27,143,394-27,143,543	192	94

S1	1GTTTTGTACTGTGGGAGTCTGAGAGCGAG	GAGGTCCGAAAGCCGAATCACAGTC2	chr19:59,386,440-59,386,493	96	-

S2	1AGCTAAAGACGTTAGGAAACAGAGCAGGGTG	GTTGAACGGGAGTGCAGCACGGTTGT2	chr19:59,396,645-59,396,701	99	-

S3	1CGACTGCGAATTACTGTTTATGAGGTGACTC	GCTGGTTCTATCGGTGGACAGTGGGACATTC2	chr19:59,386,011-59,386,072	104	-

S4	1GGTACGGAAGGCAGAATCGTACCTG	CCTTACCAGGAAAACGGACAATCTTCC2	chr21:33,785,757-33,785,822	106	14

S5	1GCCACAAACTCAAATAGGAGACTCCGC	CGGTTTTCTATTGGCTAGAGCGGAGAAGC2	chr2:234,427,935-234,428,007	115	17

S6	1ATGGGGTACGACTTCGAATCACGTGC	CCTGATGACCTCTAGAGGTAAACTCGTGCAC2	chr7:27,149,886-27,149,967	124	25

S7	1AACCTAGTCCTCCCAGGTTAGCACG	CAGCCAGCCAACGCCTCTTCTGATTG2	chr19:59,396,374-59,396,459	128	35

S8	1TGTGAGGGACCTTGTTACTGGGCAG	CATCAGGAGGTGTACTGCCGTACCATA2	chr5:131,658,970-131,659,067	140	46

S9	1CCGAGAGTGGGAGCTACTCATTTTGAGG	CCCTTTAATTAAAGTCGCAGGCACCTAGG2	chrX:153,252,787-153,252,889	145	46

S10	1CTCTGACGTAGTGTGACCTTGCTCATCC	CGAATTCAGCTCTGCTAGGACTGTTGG2	chr5:131,657,322-131,657,462	183	86

S11	1GCTCTTTGCATCGCTCTCTGTCGG	CGTCTTCGCACTTACGCGGAGCGGTAA2	chr21:33,066,198-33,066,362	207	114

	1=GGGTTCCCTAAGGGTTGGA	2=TCTAGATTGGATCTTGCTGGC			

N(n): non-sensitive, S(n): sensitive probes for HeLa cells according to data published by the ENCODE consortium. The genomic coordinates are from the human genome assembly of June 2006 (hg18).

Fragment analysis was performed on an ABI3130 capillary sequencer. Peak data was extracted using GeneMarker software (Soft Genetics) and exported to Excel (Microsoft) for further analysis.

### MLPA data analysis

Basic data analysis was performed as described [24]. Peak data for the naked DNA experiment was normalised to the average of all probes in the examined samples, with all other reactions being normalized to all non-sensitive probes. To assist in visualisation of the results, normalized ratios of all untreated samples were set to 1.

## Authors' contributions

TO and SJW conceived the experimental design. TO, SE, WNL, AHS, and SJW participated in acquisition, analysis, and interpretation of the data. TO and SJW drafted the manuscript. TO, SE, WNL, AHS, and SJW participated in critical revision and approved the final manuscript.
